# Soft tissue cysticercosis - Ultrasonographic spectrum of the disease

**DOI:** 10.4103/0971-3026.76059

**Published:** 2011

**Authors:** Deepti Naik, MG Srinath, Ashok Kumar

**Affiliations:** Department of Radiodiagnosis, MS Ramaiah Medical College and Hospital, Bangalore, India

**Keywords:** Cyst, scolex, soft tissue cysticercosis, ultrasonography

## Abstract

This case series emphasizes the role of USG in the diagnosis of isolated soft tissue cysticercosis. We assessed its value for identifying features such as the location of the cyst, the presence or absence of abscess, and the presence or absence of a scolex within the cyst. Three USG patterns were seen and are described.

## Introduction

Cysticercosis is the most common parasitic infection of the soft tissues. Cysticercosis is endemic in Mexico, Central and South America, Asia, India, sub-Saharan Africa, and China.[1–3] Due to increasing population mobility, cases of cysticercosis are now also being increasingly seen in developed countries.[3] Cysticercosis can affect various organs, including the brain, spinal cord, orbit, muscle, subcutaneous tissue, and heart.[4,5] Human beings become occasional hosts of *Taenia solium* larvae, either via drinking of contaminated water or by eating uncooked/undercooked vegetables or pork.[3] The clinical manifestations vary, depending on the site of larval encystment. USG is an inexpensive, readily available, and radiation-free modality for the diagnosis of soft tissue cysticercosis.

## Materials and Methods

Between May 2008 and October 2009, there were 17 cases of isolated soft tissue cysticercosis diagnosed solely by USG at our institute. Two cases did not come back for follow-up USG after medical treatment.

USG was performed with a Voluson-730 scanner (GE Healthcare), using a 12 MHz linear transducer. The USG findings evaluated were cyst size, shape, and margin; presence of scolex; presence of abscess/surrounding inflammation; and location of the lesion. Follow-up USG to look for resolution was performed in six patients who received medical treatment. In the other nine patients, excision and biopsy were performed due to the presence of abscess.

## Results

**Figure 1:**
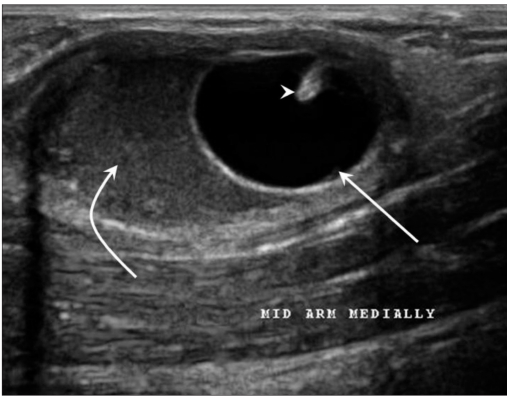
USG shows a cyst (arrow) with a scolex (arrowhead) and surrounding abscess (curved arrow)

**Figure 2:**
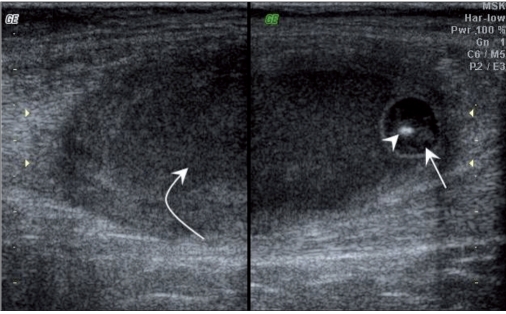
USG shows a cyst (arrow) with a scolex (arrowhead) and surrounding abscess (curved arrow)

**Figure 3:**
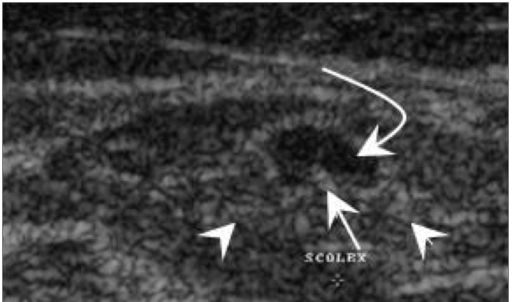
USG shows a cyst (curved arrow) with scolex (arrow) within and surrounding edema (arrowhead)

**Figure 4:**
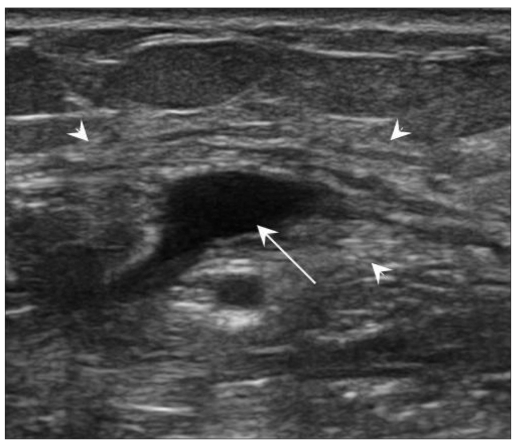
USG shows an irregular cyst (arrow) with no scolex within but with surrounding edema (arrowhead)

The most common location for soft tissue cysticercosis was in the skeletal muscles of the upper extremities [Table 1], seen in ten cases. The mean age of the patients was 28.2 years (range: 9-52 years). Out of the 15 patients, seven were vegetarian and eight were non-vegetarian. Ten of the fifteen cases were females. The most common USG appearance was that of a cyst containing a scolex within and with surrounding abscess [Figures 1 and 2]; this picture was seen in nine patients. The second most common appearance, which was seen in five patients, was that of a cyst containing a scolex within and with surrounding edema [Figure 3]. The least common appearance, which was seen in only one of the patients, was that of an irregular cyst with no scolex within and with surrounding edema [Figure 4].

**Table 1:** Demographic profile, clinical diagnosis, and sonographic features of soft tissue cysticercosis

Case#	Age (years)	Sex	Diet profile	Location of cyst	Clinical diagnosis	Ultrasonographic appearance
1	38	M	Non-vegetarian	Brachialis	Lipoma	Cyst with edema
2	20	F	Vegetarian	Deltoid	Abscess	Cyst with abscess
3	9	M	Non-vegetarian	Posterior triangle of neck (subcutaneous)	Lymphadenitis	Cyst with abscess
4	26	F	Vegetarian	Flexor digitorum profundus	Ganglion	Cyst with edema
5	43	F	Vegetarian	Biceps	Abscess	Cyst with abscess
6	27	M	Non-vegetarian	Triceps	Abscess	Cyst with abscess
7	22	F	Non-vegetarian	Masseter	Abscess	Cyst with abscess
8	52	M	Vegetarian	Rectus abdominus	Abscess	Cyst with abscess
9	14	M	Non-Vegetarian	Brachialis	Ganglion	Cyst with edema
10	25	F	Vegetarian	Vastus intermedius	Lipoma	Cyst with edema
11	18	F	Non-vegetarian	Triceps	Abscess	Cyst with abscess
12	23	F	Non-vegetarian	Latissimus dorsi	Abscess	Cyst with abscess
13	39	F	Vegetarian	Brachialis	Fat necrosis	Cystic lesion with no scolex/abscess
14	32	F	Vegetarian	Triceps	Lipoma	Cyst with edema
15	35	F	Non-vegetarian	Biceps	Abscess	Cyst with abscess

Out of the 15 patients, six were treated conservatively with albendazole or praziquantel and follow-up USG was done at 15 days and at three months. The follow-up USG done at 15 days showed resolution of edema, while the follow-up USG done at three months showed complete resolution of the cyst and edema. Nine of the fifteen patients had abscess and therefore underwent surgical excision and the diagnosis of cysticercosis was histopathologically confirmed.

## Discussion

Cysticercosis in humans is caused by consumption of food or water contaminated with viable eggs of *T. solium* or regurgitation of eggs into the stomach from the intestine of people harboring a gravid worm.[1 2] If the eggs contaminate food sources, upon ingestion they develop into larvae and result in cysticercosis.[4] Hence, even people who do not consume pork, including vegetarians, can develop cysticercosis;[1] cysticercosis is not solely caused by consumption of infested pork. Cysticercosis can affect various organs, including brain, spinal cord, orbit, muscle, and heart.[1] In this study, we have focused on isolated soft tissue cysticercosis.

High-frequency USG has become relatively inexpensive and is a readily available and reliable diagnostic modality for the diagnosis of soft tissue cysticercosis. The most common USG appearance of soft tissue cysticercosis that we encountered in this study was that of an intramuscular abscess with an eccentrically situated typical cyst with a scolex within; this picture was seen in nine patients. This appearance may be due to chronic intermittent leakage of fluid from the cyst due to degeneration of the cyst, resulting in a chronic inflammatory response with a fluid collection around the cyst.[2 3 6] The second most common appearance was that of a typical cysticercosis cyst with a scolex within and surrounding mild edema but no abscess. Such patients may present with subcutaneous nodules or pseudohypertrophy of muscles if multiple cysts are present.[2 6] The least common appearance was that of an irregular cyst with no scolex within but with minimal fluid surrounding the cyst on one side indicating leakage of fluid.[2 6] The non-visualization of the scolex may be due to escape of the scolex outside the cyst or partial collapse of the cyst during larval death.[2 6] Such patients present with myalgia.[2 6]

The clinical features depend on the location of the cyst, the cyst burden, and the host reaction.[1 4] Subcutaneous cysticercosis may cause painless or painful subcutaneous nodules.[1] Muscular cysticercosis may present clinically with myalgia, pseudotumor or mass and pseudohypertrophy.[2 6 4] Clinically, soft tissue cysticercosis can be misdiagnosed as lipoma, epidermoid cyst, abscess, pyomyositis, tuberculous lymphadenitis, neuroma, neurofibroma, sarcoma, myxoma, ganglion, or fat necrosis.[7] Since it is a common soft tissue infection, clinicians should always consider cysticercosis in the differential diagnosis whenever a patient presents with painful or painless swelling of long duration. USG is the initial and most reliable diagnostic modality for a soft tissue swelling.[8]

MRI is also used to diagnose soft tissue cysticercosis, since many patients with soft tissue swelling often go directly for MRI.[6 7 9] Cysticercosis is seen as a cystic lesion that appears hyperintense on T2W and hypointense on T1W images. Peripheral rim enhancement of the cyst wall is also known. Intramuscular cysts are oriented in the direction of the muscle fibers.[9] The scolex is also appreciated as a tiny hypointense speck within the hyperintense cyst[9] The diagnosis of cysticercosis can be confirmed by fine-needle aspiration cytology (FNAC) or biopsy, which shows the detached hooklets, scolex, and fragments of the spiral wall of *Cysticercosis cellulosae*.[8 10] Sometimes, the larval parts may not be seen in the specimen, but an inflammatory reaction consisting of large numbers of eosinophils and histiocytes can still be seen.[8]

Treatment of soft tissue cysticercosis depends on the location of the cysts.[1] Surgical excision is done for isolated skeletal muscle or soft tissue cysticercosis associated with abscess.[1 4] Cysts that are not associated with abscess can be treated with antihelminthic medications such as albendazole or praziquantel.[3 4] Follow-up USG is done after three weeks of antihelminthic medication to look for resolution of the lesion.

Cysticercosis, thus, should always be part of the differential diagnosis of subcutaneous and intra-muscular swellings in India. USG is a good modality for diagnosing soft tissue cysticercosis.
